# Is the cardiac monitoring function related to the self in both the default network and right anterior insula?

**DOI:** 10.1098/rstb.2016.0004

**Published:** 2016-11-19

**Authors:** Mariana Babo-Rebelo, Nicolai Wolpert, Claude Adam, Dominique Hasboun, Catherine Tallon-Baudry

**Affiliations:** 1 Laboratoire de Neurosciences Cognitives (ENS – INSERM U960), Département d'Etudes Cognitives, Ecole Normale Supérieure – PSL Research University, 75005 Paris, France; 2 AP-HP, Groupe Hospitalier Pitié-Salpêtrière, Paris 75013, France

**Keywords:** intracranial electroencephalography, magnetoencephalography, neural responses to heartbeats, heartbeat-evoked responses, interoception, spontaneous cognition

## Abstract

The self has been proposed to be rooted in the neural monitoring of internal bodily signals and might thus involve interoceptive areas, notably the right anterior insula (rAI). However, studies on the self consistently showed the involvement of midline default network (DN) nodes, without referring to visceral monitoring. Here, we investigate this apparent discrepancy. We previously showed that neural responses to heartbeats in the DN encode two different self-dimensions, the agentive ‘I’ and the introspective ‘Me’, in a whole-brain analysis of magnetoencephalography (MEG) data. Here, we confirm and anatomically refine this result with intracranial recordings (intracranial electroencephalography, iEEG). In two patients, we show a parametric modulation of neural responses to heartbeats by the self-relatedness of thoughts, at the single trial level. A region-of-interest analysis of the insula reveals that MEG responses to heartbeats in the rAI encode the ‘I’ self-dimension. The effect in rAI was weaker than in the DN and was replicated in iEEG data in one patient out of two. We propose that a common mechanism, the neural monitoring of cardiac signals, underlies the self in both the DN and rAI. This might reconcile studies on the self highlighting the DN, with studies on interoception focusing on the insula.

This article is part of the themed issue ‘Interoception beyond homeostasis: affect, cognition and mental health’.

## 1. Introduction

It has been proposed that the self is rooted in the neural monitoring of internal bodily signals [1,2]. For Damasio [1], for instance, the non-conscious cartography of bodily states, the ‘proto-self’, is the basis for the construction of higher level conscious forms of self, the ‘core self’ and the ‘autobiographical self’. Experimental studies of the neural bases of visceral information processing in humans have mostly relied on explicit interoception paradigms, where attention is voluntarily oriented towards internal signals and thus towards oneself. The role of the right anterior insula (rAI) in cardiac interoception has been particularly underlined, following Craig's influential theory [3] that awareness arises from the integration of visceral signals with environmental, hedonic, motivational, social and cognitive signals, in a gradient along the insular cortex, but also based on empirical findings. Indeed, both the level of activation and grey matter volume of the rAI correlate with performance in the heartbeat-counting task [4]. An involvement of insular regions during the heartbeat-counting task [5] is also compatible with the localization of the attentional modulation of heartbeat-evoked responses (HERs) [6,7]. However, the role of the rAI in explicit interoception remains debated, because interoceptive accuracy was preserved in a patient with bilateral insula damage [8]. In addition, in the heartbeat detection task, cardiac interoception modulates activity in a variety of other areas, such as somatomotor areas and the dorsal anterior cingulate cortex [4,9]. Most notably, the rAI is one of the structures most commonly activated across all cognitive tasks [10,11], and might play a more general role in switching between internally and externally oriented cognition [12].

Besides, most experimental studies of the self do not point at the insula, but at the default network (DN) [13], a network of brain regions that is more active at rest [14], during spontaneous thoughts [15] and internally directed cognition [16], than during most cognitively demanding tasks [17]. As shown in a meta-analysis [18], tasks pertaining to the *cognitive self*, such as autobiographical memory, self versus other personality trait judgement, own name detection or face recognition, consistently involve the medial nodes of the DN. This vast experimental literature does not make any explicit reference to the body or to the processing of bodily signals, and thus appears disconnected from theories relating the self to bodily signals. This overview of studies on the self and explicit interoception thus suggests the involvement of two sets of regions, the DN that is involved in the self but is not linked experimentally to bodily signals, and the rAI, that appears to be involved in the conscious perception of heartbeats.

It would logically follow that the self as expressed in the DN is not related to interoceptive signals. Still, the dichotomous view presented above has to be nuanced by a few experimental findings. First, both the DN and the rAI are found differentially activated in studies targeting the *bodily self* [19]. These studies manipulated body ownership and self-location by creating multisensory conflicts between visual and tactile information, and found a consistent involvement of the right inferior parietal lobule [20,21] and the posterior cingulate cortex (PCC) [22], i.e. two nodes of the DN, but also somatosensory regions and the insular cortex [23]. Second, the meta-analysis of the self cited above [18] focused on midline structures and showed a consistent link between midline nodes of the DN and the self, but did not draw any conclusion on the link between insula and self. Conversely, while the DN is not particularly known for being involved in autonomic regulation, we showed the existence of neural responses to heartbeats in the DN [24], which are markers of the neural processing of ascending cardiac information. We further revealed a direct link between the self and neural responses to heartbeats in the DN [25]. In a whole-brain analysis of magnetoencephalography (MEG) data, we found that the amplitude of neural responses to heartbeats in the two midline nodes of the DN (the PCC and the ventromedial prefrontal cortex, vmPFC) encoded the involvement of the self in spontaneous thoughts. These results suggest that the cardiac monitoring function of the DN is related to the neural implementation of the self.

Here, we hypothesize that a common mechanism, the neural response to heartbeats, could underlie the self in both the medial DN and the rAI. The objectives of this article are threefold. First, in a new meta-analysis of the literature, we confirm the link between the self and DN, and test the link between the self and rAI. We also probe the overlap of DN and rAI with regions involved in autonomic regulation to strengthen our proposal that visceral functions of the DN have been underestimated [25]. Second, we aim at confirming the link between neural responses to heartbeats in the DN and the self with intracranial electroencephalography (iEEG) in epileptic patients. Third, we test whether neural responses to heartbeats in the insula contribute to the self, using both iEEG and a region-of-interest (ROI) approach of the MEG data of healthy participants presented in [25].

**Figure 1.**
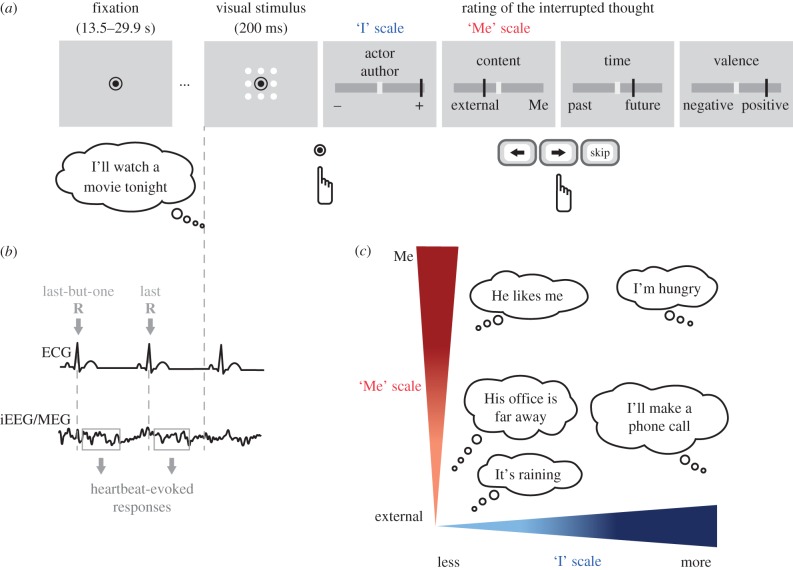
Experimental paradigm. (*a*) Time course of a trial. Each trial consisted of a fixation period interrupted by a visual stimulus. During fixation, participants were asked to let their thoughts develop freely. Participants pressed a button in response to the visual stimulus and had to remember the thought that was interrupted by the visual stimulus. They rated this thought along four scales (‘I’, ‘Me’, Time and Valence) or could skip the ratings if the interrupted thought was unclear or if they were not sure how to use the scales. (*b*) Intracranial electroencephalographic (iEEG) and magnetoencephalographic (MEG) data were locked to the two R-peaks of the electrocardiogram (ECG) preceding the visual stimulus, to compute HERs during the thought. (*c*) Examples of thoughts along the two scales of self-relatedness. The ‘I’ scale described the engagement of the participant as the protagonist or the agent in the thought. The ‘Me’ scale described the content of the thought, that can be oriented either toward oneself or toward an external object, event or person (adapted from [25]).

Both patients and healthy participants performed a thought-sampling task (figure 1*a*), where they had to fixate a point on the screen and let their mind wander freely for 13–30 s until a visual stimulus was displayed. They had to evaluate the thought they were having at the moment of stimulus display on two scales that targeted two aspects of the self (figure 1*c*). Participants evaluated on the ‘I’ scale their involvement in the thought as the subject or agent, the one who acts, feels or perceives from the first-person perspective. Ratings on the ‘I’ scale were high for thoughts such as ‘I have to make a phone call’ or ‘I am thirsty’, and low for thoughts with little engagement of the ‘I’ such as ‘It's raining’ or ‘He is coming tomorrow’. Participants evaluated on another scale to what degree they were thinking about themselves (‘Me’ scale). Ratings on the ‘Me’ scale were high when participants were thinking about themselves, such as in ‘I am thirsty’ or ‘I should be more concerned’, but low when the thought was directed towards something or someone else, as in ‘It's raining’ or ‘I will make a phone call’. We measured HERs preceding the display of the visual stimulus (figure 1*b*), and correlated the amplitude of HERs during the thought with the ratings on the ‘I’ and the ‘Me’ scales.

## 2. Material and methods

### (a) Patients

Five epileptic patients (mean age = 27.6, s.d. = 7.2; two males; right-handed; see the electronic supplementary material, table S1) gave their written informed consent to participate in this study. These patients suffered from drug-refractory focal epilepsy and were implanted stereotactically with depth electrode shafts as part of a presurgical evaluation. Implantation sites were selected on clinical criteria only, without reference to the present protocol. None of the patients had brain lesions, dysplasia nor substantial cognitive impairments. This experiment was approved by the ethics committee of Pitié-Salpêtrière Hospital (Comité de Protection des Personnes).

### (b) Intracranial electroencephalography procedure

The thought-sampling paradigm used here corresponds to the one developed by Babo-Rebelo *et al.* [25], where it is explained in full detail. Briefly, patients were presented with three to five blocks of nine trials each (electronic supplementary material, table S1). Each trial consisted of a fixation period followed by a visual stimulus. Fixations ranged from 13.5 to 29.9 s and were randomized in each block. Participants were asked to let their mind wander as naturally as possible during fixation and to press a button in response to the visual stimulus. Then, they rated the thought they were having at the moment of display of the visual stimulus, along four continuous scales. The ‘Actor/Author’ scale targeted the ‘I’ dimension of the self (‘I’ scale) and evaluated the degree to which the participant was seeing or feeling himself/herself as the actor or author during the thought. Participants were instructed to use high ratings (‘+’) when they were adopting their own perspective, i.e. when they were the protagonist or the agent of the thought, as in ‘I will make a phone call’. Low ratings (‘−’) were used when someone else was the protagonist of the thought (‘His office is far away’) or nobody in particular (‘It's raining’). The ‘Content’ scale targeted the ‘Me’ dimension of the self (‘Me’ scale), i.e. how much the thought was focused on the participant himself/herself or on something external. The ‘Me’ extreme of the scale was to be used when participants were thinking about themselves, about their feelings, body or mood, as in ‘I'm hungry’, ‘I should be more concerned’ or ‘I'm bored’. The ‘External’ extreme was to be used when participants were thinking about something that was external to them, as for instance ‘It's raining’ or ‘What was the title of the book that Peter recommended?’. The ‘Time’ scale was used to report whether the thought referred to past, present or future events, while the ‘Valence’ scale was used to determine whether the thought was pleasant or unpleasant. Participants could skip the ratings if they did not have any clear thought when the stimulus appeared or if they did not know how to rate the thought. If a trial was skipped a new one was added to the block, unbeknownst to the participant.

Before performing the actual experiment, patients were given written and oral instructions and were trained on the scales by rating five examples of thoughts. Their ratings were discussed with the experimenter to ensure task comprehension. Patients then performed a practice block of the thought-sampling task, with two trials.

### (c) Intracranial electroencephalographic data acquisition, preprocessing and electrode localization

Patients were implanted intracerebrally with 7–13 depth electrode shafts, each bearing 3–12 contacts (Ad-Tech platinum electrodes with a diameter of 1 mm and 5 mm between contacts). iEEG and electrocardiogram (ECG) data were acquired simultaneously, with either a Micromed (two patients, sampling rate: 1024 Hz; online band-pass filter: 0.15–463.3 Hz; reference: Cz electrode) or Neuralynx monitoring system (three patients; sampling rate: 4000 Hz; online low-pass filter: 1000 Hz; reference: electrode contact in the skull).

Data were downsampled to 1000 Hz and band-pass filtered off-line between 0.5 and 25 Hz, using a fourth-order Butterworth filter. All iEEG signals were re-referenced to their nearest neighbour on the same electrode shaft (bipolar montage) to limit volume-conducted influences, including the cardiac-related artefact. In the following, we will refer to these bipolar montages as ‘recording sites’.

Electrode contacts were automatically identified on the computed tomography (CT)-scan obtained after electrode implantation, using a watershed transform-based algorithm. The CT-scan and magnetic resonance imaging (MRI) obtained after implantation were registered to the pre-implantation MRI using Baladin [26], and all images were normalized to Montreal Neurological Institute (MNI) space using SPM12. The automatic electrode localization was verified visually and corrected if necessary using an interactive tool (EpiLoc toolbox developed by the STIM (Stereotaxy: Techniques, Images, Models) engineering platform (http://icm-institute.org/en/cenir-stim-stereotaxy-core-facility-techniques-images-models-2/) in the Institut du Cerveau et de la Moelle Epinière, in Paris). The coordinates of each recording site are reported as the coordinates of the midpoint between the two corresponding contacts.

### (d) Rationale for intracranial electroencephalographic analyses

iEEG analyses were restricted to a subsample of recording sites selected on the basis of their distance to the regions where previous MEG results [25] were found (posteromedial cortex, vmPFC) or where we defined *a priori* ROIs (insula). For each region, we defined a volume of interest as the union between the MEG cluster and the corresponding functional territories. For instance in posteromedial cortex, we considered the voxels in the MEG cluster as well as the voxels belonging to the ventral precuneus and ventral posterior cingulate cortex (vPCC). We selected recording sites inside, or at less than 6 mm from the borders of this volume. This limit of 6 mm corresponds to a fair approximation of the borders of these regions, considering both the smoothness applied to functional MRI and MEG source localization masks, but also considering the accuracy of bipolar intracranial recordings.

Even though all patients responded to all scales at each trial, we only analysed the data corresponding to the scale of interest given the MEG results. Therefore, only the ‘I’ scale was analysed for recording sites in the posteromedial cortex (patient 4) and in the insular region (patients 3 and 5), and only the ‘Me’ scale was analysed for recording sites in the vmPFC (patients 1, 2 and 3).

### (e) Intracranial heartbeat-evoked responses analysis

To detect R-peaks in the ECG, we correlated the *z*-scored ECG signal with a template QRS complex created for each patient and identified the local maxima within episodes of correlation larger than a threshold chosen for each patient. R-peak detection was verified by checking for the absence of outliers in the interbeat-interval distribution as well as by visual inspection in a time window from −6 to 3 s relative to the visual stimulus.

Epochs of iEEG data were extracted from −100 to 600 ms relative to the two R-peaks preceding each visual stimulus by at least 700 ms. Epochs that exceeded ±200 mV, which showed a dynamic range of 300 mV or more in a 20 ms interval were excluded from analysis. Data were subsequently visually inspected to discard any additional epochs with excessive noise or epileptic activity. Because recording sites of interest were far from epileptic foci, we discarded only a few epochs (less than 14.8% of the trials in all patients). The final number of trials used in the analysis is reported in the electronic supplementary material, table S1. For each trial, we averaged the two obtained epochs, resulting in one HER per trial and per recording site of interest.

We aimed at testing for each recording site whether the amplitude of HERs was modulated by the self-relatedness of ongoing thoughts. For each time point *t* of the HER, we computed across trials the Pearson correlation between the *z*-scored HER amplitude at time *t* and the corresponding *z*-scored rating of the thought on the scale being tested. We then obtained a time course of Pearson correlations and a time course of *t*-values of the Pearson correlation, revealing the amount of correlation between HER amplitude and ratings on the scale of interest at each time point of the HER. We here used a correlational approach at the single trial level rather than comparing the average HERs for trials rated as high and for trials rated as low as in MEG data [25] to take advantage of the higher signal-to-noise ratio of the iEEG data.

To look for time windows where HER amplitude significantly correlates with ratings, while correcting for multiple comparisons over the time domain, we applied a cluster-based permutation test [27] on the two-tailed *t*-values of Pearson's correlation across time samples of the time window 300–600 ms relative to the R-peak, for each recording site. Briefly, individual samples with a *t*-value corresponding to a *p*-value below an arbitrarily selected threshold (*p* < 0.05, two-tailed) are clustered together based on temporal adjacency. Clusters are characterized by the sum of *t*-values of the individual samples. To establish the likelihood that a cluster was obtained by chance, we shuffled 10 000 times the ratings with respect to the HERs and repeated the clustering procedure selecting the maximum positive cluster-level statistic and the minimum negative cluster-level statistic. The Monte Carlo *p*-value corresponds to the proportion of elements in the distribution of maximal (or minimal) cluster-level statistics that exceeds (or is inferior to) the originally observed cluster-level test statistics and is intrinsically corrected for multiple comparisons on time samples. The statistical tests were restricted to the time window 300–600 ms post R-peak and not to the entire HER, because this time window is known to be devoid of the cardiac-field artefact [28]. We also applied a Bonferroni correction on the Monte Carlo *p*-values, to account for the number of recording sites tested per patient.

Note that here HERs were locked to R-peaks, not to T-peaks as in [25], because T-peaks could not be reliably identified on the ECG signal that had a lower signal-to-noise ratio in clinical settings. To compare latencies between the previous MEG [25] results and the results presented in the current paper both in MEG and iEEG, one has to keep in mind that the average R-T interval in the MEG data is 269 ms.

### (f) Surrogate heartbeats

To demonstrate that the observed effects were locked to heartbeats, we checked whether the correlations between HER amplitude and ratings could be obtained with the same sampling of the neural data but unsynchronized with heartbeats. We created 1000 permutations of heartbeats, where the timing of the pair of heartbeats of trial *i* in the original data is randomly assigned to trial *j*. The same criteria for rejecting artefactual epochs and computing of HERs was applied. For each permutation, we obtained a set of neural responses to surrogate heartbeats and computed the cluster summed *t* statistics as described above. For each permutation, we extracted the smallest sum of *t*-values for recording site 2 of patient 1 and recording site 1 of patient 4 (because the original sum of *t*-values was negative), and the largest sum of *t*-values for the recording site 2 of patient 3 (because the original sum of *t*-values was positive). We then compared the distribution of those surrogate values with the observed original sum of *t*-values. This control was performed on iEEG data (for MEG data, see [25]).

### (g) Region-of-interest analysis on magnetoencephalographic data

We here used an ROI approach centred on the insula, to analyse the MEG data of Babo-Rebelo *et al.* [25]. Sixteen healthy participants (mean age: 24.1 ± 0.6 yr, eight males) performed five blocks of 16 trials of the thought-sampling task, while MEG activity (Elekta Neuromag TRIUX with 102 magnetometers and 204 gradiometers, sampling rate of 1000 Hz, online low-pass filtered at 330 Hz) was acquired simultaneously with ECG activity (seven electrodes around the neck, 0.03–330 Hz). MEG and ECG data were band-pass filtered between 0.5 and 25 Hz. The cardiac-field artefact was corrected on the MEG data using an independent component analysis. HERs were obtained at the sensor level by averaging brain activity locked to the two R-peaks preceding the visual stimulus. For each scale, trials were median split and an average HER was computed for trials rated as ‘high’ and for trials rated as ‘low’. We here used a median split approach because analyses were done at the group level, on data that has a lower signal-to-noise ratio compared with iEEG. Source localization of the HERs was performed with the BrainStorm toolbox [29], using a model consisting of 15 002 current dipoles from the combined time series of magnetometer and gradiometer signals using a linear inverse estimator (weighted minimum-norm current estimate). We created BrainStorm scouts using the niftii masks from Deen *et al.* [30], to identify the vertices corresponding to the three right insular ROIs: posterior insula (PI, 50 vertices), ventral anterior insula (vAI, 82 vertices) and dorsal anterior insula (102 vertices). Dorsal and vAI scouts had 33 vertices in common, owing to the low resolution of the source model relative to the MRI masks. We then averaged the neural currents corresponding to each of the scouts, and compared, for each ROI, the average cortical current corresponding to ‘high’ ratings with the one corresponding to ‘low’ ratings, on the ‘I’ and ‘Me’ scales separately. To assess the statistical difference in HERs between ‘high’ and ‘low’ ratings, while controlling for multiple comparisons over the time domain, we applied as before a cluster-based permutation test for each ROI, but based on the *t*-test between ‘high’ and ‘low’ conditions. The resulting Monte Carlo *p*-values were Bonferroni corrected for testing on two different scales (‘I’ and ‘Me’).

We also tested for a correlation between ROI results and individual interoceptive abilities, which were measured in the 16 MEG participants using the heartbeat-counting task [5], over six blocks of variable durations (30–120 s) [25]. Interoceptive abilities were not measured in patients.

### (h) Meta-analysis of the ‘self’

This meta-analysis was performed using the Neurosynth platform (http://neurosynth.org) [31] that contains nearly 11 400 neuroimaging studies (May 2016). From each article, Neurosynth automatically extracts a set of terms that occur at a high frequency (greater than 1 in 1000 words) and the activation coordinates reported in the study (coordinates are transformed to MNI space if necessary). The database currently contains 3107 terms. We explored the term ‘self’, which appeared in 903 studies and encompassed 33 560 activations. The automated meta-analysis corresponds to a statistical inference map, from the comparison of coordinates reported in studies containing the term ‘self’ with coordinates from studies that do not contain the term. The forward inference map corresponds to *z*-scores of the likelihood that a voxel will be activated if a study uses the term ‘self’ (P(Activation|Term)). The forward inference map thus corresponds to regions that are *consistently* active in studies related to the self, but that may also be active in other paradigms not related to the self. The reverse inference map reports the *z*-scores corresponding to the likelihood that ‘self’ is used in a study given the presence of reported activation in a particular voxel (P(Term|Activation)). The reverse inference map therefore corresponds to regions that are *selectively* associated with the word ‘self’. The reverse inference map controls for base rate differences between regions, so regions that lack selectivity (i.e. regions that are associated with many different terms) are not included in the map. Both maps were corrected for multiple comparisons using a false-discovery rate (FDR) approach, with an FDR of 0.01, meaning that about 1% of activated voxels are false positives, as intrinsically implemented in the Neurosynth platform.

### (i) Overlap between our results and anatomical parcellations and meta-analyses

We used a structural MNI152 template image on mricron (https://www.nitrc.org/projects/mricron) to represent our results and the overlap with parcellations and meta-analyses. The MNI coordinates of the vertices showing significant differential HER activity in a previous MEG study [25] were obtained using the BrainStorm functions cs_scs2mri and cs_mri2mni. Niftii masks were then created, displaying the significant voxels, that were expanded (we considered a square of three voxels side, centred on the significant voxel) to facilitate visualization. These masks were then overlaid with the parcellation of the posteromedial cortex from [32], the parcellation of the vmPFC from [33] and the parcellation of the insula from [30]. We also overlaid all results with masks resulting from a meta-analysis on the autonomic brain as described in [34]. All masks were transformed to a final dimension of 91 × 109 × 91, using the function ImCalc of SPM12. The masks of the posteromedial cortex [32], the vmPFC [33], the insula [30] and of the autonomic brain [34] were provided by the corresponding authors.

## 3. Results

### (a) The self and autonomic regulation

**Figure 2.**
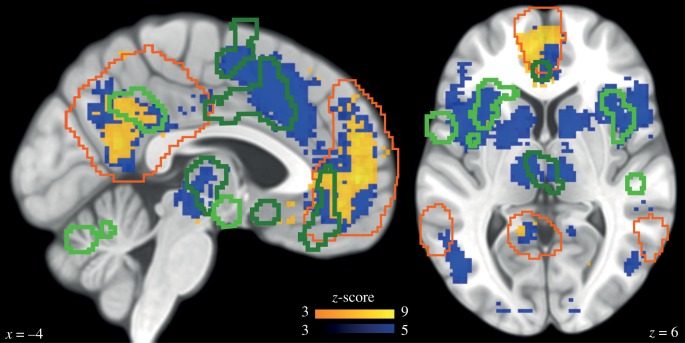
Overlap between DN, self and autonomic regulation meta-analyses. The orange outline represents the DN, as defined in Laird [35]. Green outlines highlight regions responsible for sympathetic (dark green) and parasympathetic (light green) regulation [34]. The results of the automated [31] meta-analysis on the term ‘self’ are presented in yellow (reverse inference map) and in blue (forward inference map). The sagittal view (left) shows that the reverse inference map of the self is associated with the DN, where it overlaps with autonomic regulation regions. The axial view (right) shows that the rAI is associated with the forward inference map of the self and overlaps with autonomic regulation regions.

To evaluate the contribution of the DN and rAI to the self, as well as their overlap with regions involved in autonomic regulation, we first conducted an automated meta-analysis [31] of 903 studies pertaining to the self. This analysis confirms on a large dataset that the DN is *selectively* related to the self (figure 2; electronic supplementary material, table S2, reverse inference): activity in the DN is likely to indicate self-related processing. The insula is *consistently* activated in the literature related to the self (figure 2; electronic supplementary material, table S3, forward inference), but is not *selective* of the self. In other words, differential activation in the insula can pertain to the self but can also be found in many other cognitive paradigms.

Regions associated with autonomic regulation [34] overlap with self-related regions in the rAI, but also in the DN: the posterior midline node of the DN is associated with parasympathetic regulation, while the frontal midline node of the DN is associated with sympathetic regulation (figure 2).

### (b) The ventromedial prefrontal cortex and the ‘Me’

In a previous MEG study [25], we found that the amplitude of HERs correlates with the involvement of the ‘Me’ dimension in the left vmPFC. A further analysis of the MEG cluster showed that it is located mainly in areas 14 m and 32 of the medial frontal cortex (table 1), according to the anatomical parcellation of Neubert *et al.* [33]. Moreover, 41.3% of the MEG cluster overlapped with sympathetic regulation regions (derived from studies on electrodermal activity) [34] which were also mainly located in areas 14 m and 32 (table 1).

**Table 1.** Percentage distribution of the anterior MEG cluster and of the sympathetic regulation areas [34] on the different sub-regions of the vmPFC [33]. The remaining 29% of the MEG cluster was located in the undetermined territory lying in between those three regions.

	14 m only	32 only	overlap 14 m and 32	11 m
MEG cluster (%)	29	28	4	10
sympathetic regulation areas (%)	46	22	4	5

To try and replicate the MEG results with intracranial recordings, we selected recording sites inside 14 m or 32 regions or at less than 6 mm from the borders of these regions (electronic supplementary material, table S4). We therefore analysed three recording sites on the left hemisphere from two different patients. We tested each recording site for a trial-by-trial correlation between the amplitude of HERs and the ratings on the ‘Me’ scale.

Trial-by-trial HERs were obtained by averaging brain activity locked to the two R-peaks preceding each visual stimulus. We computed at each time point the Pearson's correlation across trials between HER amplitude and the rating of the thought on the ‘Me’ scale. We then used a clustering procedure, which corrects for multiple comparisons over time, to identify, within the time window 300–600 ms after the R-peak, moments where HER amplitude significantly correlated with ratings on the ‘Me’ scale.

**Figure 3.**
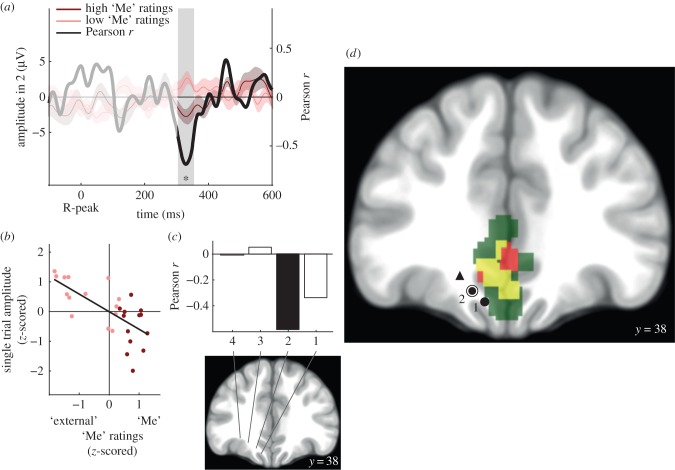
The trial-by-trial amplitude of HERs in the vmPFC correlates with the involvement of the ‘Me’ in spontaneous thoughts (patient 1). (*a*) Time course of the Pearson correlation coefficient *r* between the trial-by-trial HER amplitude and the ratings on the ‘Me’ scale (black), and HERs (±s.e.m.) for ‘high’ (dark red) and ‘low’ (light pink) ratings on the ‘Me’ scale (median split of ratings), for recording site 2 (circled dot in (*d*)). The signal that might be residually contaminated by the cardiac-field artefact appears in lighter colour (not included in the analysis). The grey area highlights the time window in which a significant trial-by-trial correlation between HER amplitude and ‘Me’ ratings was observed. (*b*) HER amplitude in the significant time window plotted against ‘Me’ ratings. Each point represents one trial. (*c*) Mean Pearson correlation coefficient in the 304–354 ms time window, along the different recording sites of the electrode shaft of patient 1. The black bar corresponds to the recording site for which a significant correlation was found. (*d*) Differential HERs, sympathetic regulation and vmPFC. Recording site 2 (circled dot) showed the significant correlation, while recording site 1 and the triangle (patient 2) showed no effect. Regions in red showed differential responses to heartbeats along the ‘Me’ scale, in a previous MEG study [25]. Regions in green are involved in sympathetic regulation [34]. Yellow corresponds to the overlap between MEG results and sympathetic regulation regions.

We found that the amplitude of HERs in recording site 2 of patient 1 (MNI coordinates: −14 38 −16, figure 3*d*) significantly correlated with ‘Me’ ratings (cluster sum(*t*) = −9547, Monte Carlo *p* = 0.046, Bonferroni-corrected for the two recording sites tested in patient 1), in the time window 304–354 ms after the R-peak (mean Pearson correlation coefficient = −0.58; figure 3*a,b*). The mean Pearson correlation coefficient in this time window decreased at recording sites that were further away from the midline (figure 3*c*). To show that the observed effects were truly locked to heartbeats and not driven by slow fluctuations of neural activity, we created 1000 permutations of surrogate heartbeats and performed the same analyses on the recording site 2 of patient 1. Only three permutations generated a cluster *t* statistic exceeding the original one (Monte Carlo *p* = 0.003; electronic supplementary material, figure S2*a*), confirming that these results are indeed locked to heartbeats.

We also tested for a correlation between HER amplitude and ‘I’ ratings at recording site 2 of patient 1, and found a significant correlation (cluster sum *t* = −8424, Monte Carlo *p* = 0.0328, uncorrected, cluster time window: 306–352 ms after the R-peak). This is different from the group-level analysis of MEG data that revealed a specific effect for the ‘Me’ in vmPFC [25]. It should be noted that in patient 1, the correlation of the ratings between the ‘I’ and the ‘Me’ dimensions was very high (Pearson *r* = 0.91), higher than in other patients (electronic supplementary material, table S1) or healthy participants (electronic supplementary material, table S7). iEEG results in this patient thus confirm that neural responses to heartbeats in vmPFC covary with the self, but do not bring any further information on the dissociation between the ‘I’ and ‘Me’ dimensions.

According to the individual anatomy of patient 1 (electronic supplementary material, figure S1*a*), the recording site 2 was located in between the cingulate sulcus, where MEG results were found, and the olfactory sulcus. Recording site 1 of patient 1, that was located more ventrally in the olfactory sulcus (electronic supplementary material, figure S1*a*), did not show any significant correlation (Monte Carlo *p* = 0.68, Bonferroni-corrected for the two recording sites tested in patient 1). In patient 2, a recording site located not in the vicinity of the medial wall but more laterally in the orbitofrontal cortex (fundus of the intermediate orbital sulcus, electronic supplementary material, figure S1*b*) did not show any significant correlation either (no candidate clusters). Altogether, the pattern of results observed with intracranial data is compatible with a neural source in the cingulate sulcus, which is included in the MEG cluster.

MEG results further suggest that HERs in vmPFC are left lateralized. We tested for a null effect at a recording site in the right homologue 14 m region, from a different patient (patient 3). This contact, located in between the olfactory sulcus and the supraorbital sulcus (electronic supplementary material, figure S1*c*), did not show any significant effects (no candidate clusters). This iEEG negative result in the right hemisphere is compatible with the left-lateralization of self-related HERs in vmPFC observed in MEG, but might also be due to an electrode location too ventral to pick activity from the cingulate sulcus and gyrus. Note that a significant effect was observed in this patient at a different location, as described below, indicating that this patient understood the task.

No correlation between heart rate and ‘Me’ ratings was observed (Pearson correlation between ‘Me’ ratings and the interval between the two R-peaks preceding the visual stimulus: *r* = 0.12, *t*_25_ = 0.58, *p* = 0.57).

The results of iEEG data thus confirm the existence of HERs distinguishing between different levels of self-relatedness of spontaneous thoughts on the ‘Me’ scale in the cingulate sulcus, at the border between areas 32 and 14 m as identified with MEG. Regions located more ventrally or more laterally did not show the effect. The areas involved respond to heartbeats and thus seem to be monitoring visceral inputs, but they are also involved in sympathetic regulation.

### (c) The posteromedial cortex and the ‘I’

**Figure 4.**
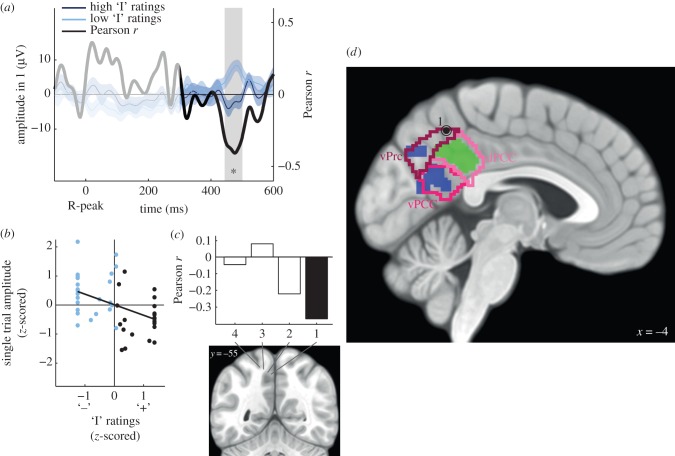
The trial-by-trial amplitude of HERs in the ventral precuneus and vPCC correlates with the involvement of the ‘I’ in spontaneous thoughts (patient 4). (*a*) Time course of the Pearson correlation coefficient *r* between the trial-by-trial HER amplitude and the ratings on the ‘I’ scale (black), and HERs (±s.e.m.) for ‘high’ (dark blue) and ‘low’ (light blue) ratings on the ‘I’ scale (median split of ratings), for recording site 1. The signal that might be residually contaminated by the cardiac-field artefact appears in lighter colour (not included in the analysis). The grey area highlights the time window in which a significant trial-by-trial correlation between HER amplitude and ‘I’ ratings was observed. (*b*) HER amplitude in the significant time window plotted against ‘I’ rating. Each point represents one trial. (*c*) Mean Pearson correlation coefficient in the 444–500 ms time window, along the different recording sites of the electrode shaft. The black bar corresponds to the recording site for which a significant correlation was found. (*d*) Differential HERs, parasympathetic regulation and posteromedial cortex. The circled dot indicates the location of recording site 1. Regions in blue showed differential responses to heartbeats along the ‘I’ scale, in a previous MEG study [25]. Regions in green are involved in parasympathetic regulation [34]. Outlines correspond to the parcellation of the posteromedial cortex [32]: ventral precuneus (vPrc, dark pink), dorsal cingulate (dPCC, light pink) and ventral cingulate cortex (vPCC, pink).

In MEG data [25], HER amplitude in the left posteromedial cortex was shown to correlate with the involvement of the ‘I’ in ongoing spontaneous thoughts. By comparing the MEG cluster with the anatomical parcellation of the posteromedial cortex by Bzdok *et al*. [32], we here show that 50.4% of the MEG cluster was located in the left vPCC and 31.5% in the left ventral precuneus (vPrc) (table 2 and figure 4*d*). Interestingly, none of these regions seems to be involved in parasympathetic regulation (mostly derived from high-frequency heart rate variability [34]), which is exclusively associated with the dorsal PCC (figure 4*d* and table 2).

**Table 2.** Percentage distribution of the posterior MEG cluster and of the parasympathetic regulation areas [34] on the different sub-regions of the posteromedial cortex [32]. The remaining 18% of the MEG cluster were located more posteriorly, in the vicinity of the parieto-occipital sulcus and calcarine fissure.

	precuneus	ventral posterior cingulate cortex	dorsal posterior cingulate cortex	retrosplenial cortex
MEG cluster (%)	32	50	0	0
parasympathetic regulation areas (%)	6	0	92	0

In order to confirm the involvement of the ventral precuneus territory, we analysed two recording sites of patient 4, which were inside the left vPrc or at less than 6 mm from its borders (electronic supplementary material, table S5). We tested for a trial-by-trial correlation between the amplitude of HERs in these recording sites and the ratings on the ‘I’ scale, in accordance with the MEG results.

We found that the amplitude of HERs recorded in the most medial recording site that was located inside the vPrc region (figure 4*d*; electronic supplementary material, figure S1*d*, recording site 1, MNI coordinates: −3 −53 49) significantly correlated with ‘I’ ratings (cluster sum(*t*) = −8395, Monte Carlo *p* = 0.041, Bonferroni-corrected for the two recording sites tested in patient 4) in the time window 444–500 ms after the R-peak (mean Pearson correlation coefficient = −0.37) (figure 4*a,b*). Recording site 2, that was located just outside the vPrc region, did not show a significant correlation with ‘I’ ratings (Monte Carlo *p* = 0.38, Bonferroni-corrected for the two recording sites tested in patient 4). More generally, the average Pearson correlation coefficient in the 444–500 ms time window decreased as we tested recording sites from the same electrode shaft that were further away from the midline (figure 4*c*). The test on the 1000 permutations of surrogate heartbeats on recording site 1 confirmed that the effects were truly locked to heartbeats (Monte Carlo *p* = 0.011; electronic supplementary material, figure S2*b*). Additionally, we did not observe a correlation between HER amplitude and ‘Me’ ratings at recording site 1 (no candidate clusters), nor between the cardiac rhythm and ‘I’ ratings (Pearson correlation between the interval between the two R-peaks preceding the visual stimulus and ‘I’ ratings: *r* = −0.13, *t*_43_ = −0.83, *p* = 0.41).

Taken together, the MEG and iEEG results from one patient consistently indicate that two sub-regions of the posteromedial cortex, the vPrc and the vPCC, respond differentially to heartbeats depending on the involvement of the ‘I’ in thoughts, whereas the adjacent dorsal PCC is involved in cardiac rate regulation but not in encoding self-related information.

### (d) The right insula and the ‘I’

**Figure 5.**
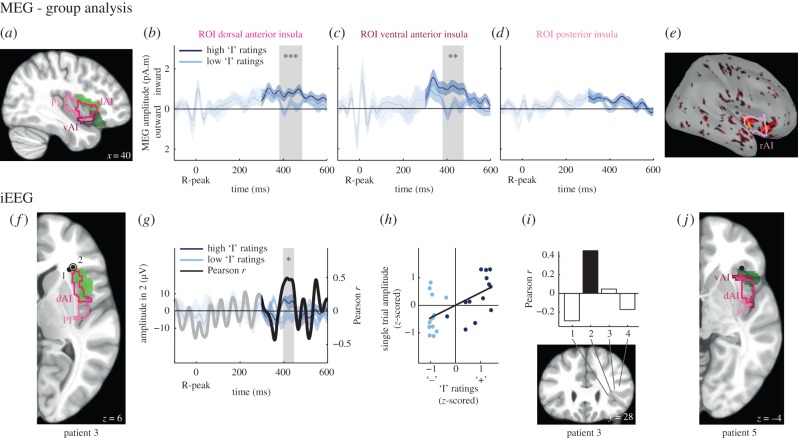
The amplitude of HERs in the rAI correlates with the involvement of the ‘I’ in spontaneous thoughts, in MEG (panels *a–e*) and in iEEG (panels *f–j*). (*a*) Sagittal view of the insula with three insular sub-regions [30] highlighted: PI (light pink), dAI (pink), vAI (dark pink). Light green and dark green regions are associated with parasympathetic and sympathetic regulation respectively [34]. (*b–d*) Time course of the HER (±s.e.m. across the 16 participants) for ‘high’ and ‘low’ responses on the ‘I’ scale (median split of responses), for the three ROIs in MEG source analysis. The grey area highlights the time window where a significant difference between HERs for ‘high’ and ‘low’ ratings on the ‘I’ scale was observed. (*e*) Differential MEG source activity for ‘high’ versus ‘low’ ratings on the ‘I’ scale averaged over 384–480 ms post R-peak (threshold for visualization: uncorrected *p* < 0.05; 75% smoothness applied to the cortical surface). The pink region corresponds to the rAI (union of dAI and vAI). (*f*) Axial view of the right hemisphere showing the PI (light pink) and dAI (pink). Black dots correspond to the two recording sites analysed in patient 3. Recording site 2 (circled dot) showed a significant correlation between HER amplitude and ‘I’ ratings, in a time window consistent with the MEG results. Areas in green are involved in parasympathetic regulation [34]. (*g*) Time course of the trial-by-trial Pearson correlation coefficient *r* between HER amplitude and ‘I’ ratings (black), and HERs (±s.e.m) for ‘high’ (dark blue) and ‘low’ (light blue) ratings on the ‘I’ scale (median split of responses), for recording site 2 of patient 3 (circled dot in *f*). The grey area highlights the time window in which a significant correlation between HER amplitude and ‘I’ ratings was observed. (*h*) HER amplitude in the significant time window plotted against ‘I’ ratings. Each point represents one trial. (*i*) Mean Pearson correlation coefficient in the 397–443 ms time window, along the different recording sites of the electrode shaft of patient 3. (*j*) Axial view of the right hemisphere showing the PI (light pink) and dAI (pink), for patient 5. The black dot corresponds to the recording site analysed for this patient, where no significant correlation was observed.

The insula can be sub-divided in three distinct regions: PI, dorsal anterior (dAI) and vAI [30]. Both right dAI and vAI regions are involved in autonomic regulation (figure 5*a,e,f,g* and table 3), as shown by the meta-analysis by Beissner *et al*. [34]. While parasympathetic regulation is uniquely associated with the dAI, sympathetic regulation is equally associated with dAI and vAI (table 3). Even though the PI is a known visceral centre of the brain, it did not appear to be associated with either sympathetic or parasympathetic regulation (table 3). Here, we test whether HER amplitude covaries with self-relatedness, first by an ROI analysis of the MEG data on healthy participants, and then by analysing three iEEG recording sites in the vicinity of the insula.

**Table 3.** Percentage distribution of insular sympathetic and parasympathetic regulation areas [34] on the different sub-regions of the insular cortex [30]. The sympathetic and parasympathetic insular regions extended over a larger area than the insular parcellation [30].

	dorsal anterior insula	ventral anterior insula	posterior insula
parasympathetic regulation (%)	29	0	1
sympathetic regulation (%)	8	8	0

#### (i) Region-of-interest analysis of the insula in magnetoencephalographic data of healthy participants

From MEG data obtained in 16 healthy participants, we computed R-locked HERs and the corresponding sources for trials rated as ‘high’ and trials rated as ‘low’ (median split of the trials, electronic supplementary material, table S7) on each self-related scale. We then averaged the resulting neural currents for the vertices belonging to each sub-region of the insula, the PI, the dAI and the vAI, according to the parcellation of Deen *et al*. [30] (figure 5*a*). For each sub-region, we searched for time windows where HERs significantly differed between trials rated as ‘high’ and trials rated as ‘low’, separately on the ‘I’ and the ‘Me’ scales, using a cluster-based permutation *t*-test over the time window 300–600 ms post R-peak.

We found that neural responses to heartbeats in the dorsal and ventral rAI (figure 5*b,c*) significantly differed for trials rated as ‘high’ and trials rated as ‘low’ on the ‘I’ scale (dAI: cluster sum(*t*) = −296, Monte Carlo *p* = 8 × 10^−4^; vAI: cluster sum(*t*) = −283, Monte Carlo *p* = 0.0012, Bonferroni-corrected for the two scales tested), in the same time window relative to the R-peak (dAI: 384–486 ms; vAI: 384–480 ms). No differences were observed in the PI (figure 5*d*, no candidate clusters) nor for the ‘Me’ scale in any of the three right insular regions (dAI: Monte Carlo *p* = 0.13; vAI: Monte Carlo *p* = 0.33; PI: Monte Carlo *p* = 0.16, Bonferroni-corrected for the two scales tested). This ROI-based approach in MEG sources thus revealed differential neural responses to heartbeats in the rAI depending on the involvement of the ‘I’ in thoughts. The map of the *t*-values associated with the ROI effect (figure 5*e*) shows that there are two foci contributing to the rAI effect, one more posterior and another one more anterior, extending outside the rAI into the inferior frontal gyrus.

We then tested the lateralization of this result, by probing the left dorsal and left vAI. No significant differences between ‘high’ and ‘low’ ratings on the ‘I’ scale were observed (all Monte Carlo *p* > 0.3). In addition, an ANOVA on brain currents averaged over the time window of the significant difference, with hemisphere (left and right) and condition (‘high’ and ‘low’) as factors revealed an interaction between hemisphere and condition in both dAI and vAI (dAI: interaction: *F*_1,15_ = 7.67, *p* = 0.014, main effects: *p* > 0.14; vAI: interaction *F*_1,15_ = 7.73, *p* = 0.014, main effect hemisphere: *F*_1,15_ = 4.72, *p* = 0.046, main effect condition: *F*_1,15_ = 2.04, *p* = 0.17). The amplitude of the effects was not modulated by individual interoceptive abilities (Pearson correlation between the difference in HER amplitude between ‘high’ and ‘low’ ‘I’ ratings and interoceptive scores, dAI: mean *r* = 0.08, *t*_14_ = 0.3, *p* = 0.8; vAI: mean *r* = −0.03, *t*_14_ = −0.1, *p* = 0.9).

#### (ii) Intracranial electroencephalographic analysis of three recording sites in the vicinity of the insula

We then analysed the iEEG data from two patients (3 and 5) who had recording sites at less than 6 mm of the borders of the right dAI (electronic supplementary material table S6). Because MEG results indicated a link between rAI and the ‘I’ scale, we searched for a trial-by-trial correlation between the HER amplitude at these recording sites and the ratings on the ‘I’ scale. The clustering test revealed a significant correlation between HER amplitude and ‘I’ ratings, at the most dorsal recording site (recording site 2 in patient 3; figure 5*f*), at a latency of 397–443 ms after the R-peak (figure 5*g,h*; Pearson correlation coefficient = 0.46, cluster sum(*t*) = 5609, Monte Carlo *p* = 0.014, Bonferroni-corrected for the two sites tested in this patient). The significant time window in iEEG data from this site is included in the time window where significant effects are found in MEG data. Moreover, the mean Pearson correlation coefficient in this time window decreased for recording sites that were located further away from the insular cortex (figure 5*i*). The result was truly locked to heartbeats (Monte Carlo *p* = 0.001, electronic supplementary material, figure S2*c*). Additionally, HER amplitude did not correlate with ‘Me’ ratings, for recording site 2 of patient 3 (Monte Carlo *p* = 0.48, uncorrected). Last, these results were not associated with a correlation between heart rate and ‘I’ ratings (Pearson correlation between ‘I’ ratings and the interval between the two R-peaks preceding the visual stimulus: *r* = −0.05, *t*_24_ = −0.25, *p* = 0.80).

The recording site where we found a significant effect was located at the anterior and dorsal border of the dAI (figure 5*f*; electronic supplementary material figure S1*e*). The other recording site in the same patient was located more ventrally and did not show any significant effect (recording site 1: Monte Carlo *p* = 0.53; Bonferroni-corrected for the two recording sites tested in patient 3). The recording site of patient 5 was located even more ventrally and did not display any significant correlation (Monte Carlo *p* = 0.50 figure 5*j*; electronic supplementary material, figure 1*f*).

iEEG data thus only partially confirm MEG results, with positive results in one patient out of two. Still, the pattern of results in both MEG and iEEG indicate that at least in its most anterior and dorsal part, the rAI generates HERs, the amplitude of which depends on the involvement of the ‘I’ in spontaneous thoughts.

### (e) Comparison of magnetoencephalographic results across vPrc/vPCC, vmPFC and rAI

Here, we used an ROI analysis to show that the rAI is differently responding to heartbeats depending on the self-relatedness of thoughts. However, the rAI did not appear in the whole-brain analysis, as opposed to the midline regions of the DN, the vPrc/vPCC and the vmPFC. We thus attempted at characterizing further the effects in rAI, to understand why this effect was not present in the whole-brain analysis.

**Figure 6.**
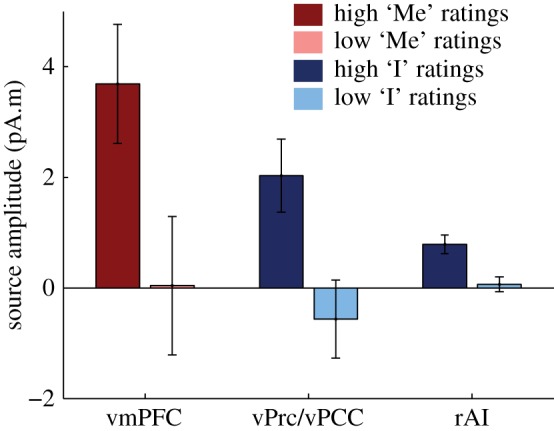
Comparison of effects in the insula and in the DN. Source activity was averaged for each significant time window, across the significant vertices (vmPFC: left, vPrc/vPCC: middle) or across the vertices belonging to the rAI ROI (right).

We first looked at effect sizes (figure 6). We averaged source amplitudes separately in the vmPFC and vPrc/vPCC clusters derived from the whole-brain analysis, and in the rAI region, defined as the union of dAI and vAI that both showed an effect in the ROI-based approach. Effect size is 3.6 times smaller in rAI than in the vPrc/vPCC and five times smaller than in the vmPFC. Effect size comparison remains difficult to interpret because voxels were selected on the basis of a statistical threshold in vmPFC and vPrc/vPCC, while voxels in the AI were selected based on an anatomically defined ROI, which includes non-responsive regions (figure 5*e*). We thus compared source amplitude at vertices thresholded at first-level *p* < 0.01 in the rAI, vPrC/vPCC and vmPFC. Effect size remained 1.7 times smaller in rAI than in the vPrC/vPCC and 2.4 times smaller than in vmPFC.

Another reason why the rAI effect was not picked up in the whole-brain analysis is that the clustering procedure employed favours spatial contiguity. As shown in figure 5*e*, it seems that there are two separate sub-regions of the rAI responding differentially to heartbeats, one in the posterior part of the rAI, another one in the anterior part, extending anteriorly in the inferior frontal gyrus.

Overall, our results indicate that the regions showing the most consistent modulation of HER amplitude in relation to the self are the midline nodes of the DN. The rAI appears to be also involved, but to a lesser extent.

## 4. Discussion

We aimed at confirming and specifying the existence of visceral monitoring functions in the DN and their links with the self, and at testing whether this mechanism could also be at play in the rAI. We first showed that both the DN and the rAI include regions involved in autonomic functions [34]. We confirm the link between the DN and self [18] and show further that the DN is specific to the self, as opposed to the rAI that is associated with the self, but also with many other, non-self-related paradigms. We found that in two patients the trial-by-trial amplitude fluctuations of intracranially recorded HERs in the DN covaried with the trial-by-trial measure of the involvement of the self in spontaneous thoughts, confirming and refining previous MEG results [25]. An ROI approach of the rAI revealed that both in MEG data of healthy participants and in intracranial recordings of one patient out of two, neural responses to heartbeats covaried with the ‘I’ dimension of the self. None of these results were associated with changes in heart rate.

## 5. Methodological considerations and limitations

In this study, we combine data from different sources. The MEG source localization results obtained in a group of healthy participants might be spatially inaccurate, but participants could be trained and task comprehension could be tested and quantified. iEEG data have high spatial accuracy and good signal-to-noise ratio, but are obtained in patients. In patients, task comprehension was not tested beyond verbal exchanges with the experimenter. Patient 1 for instance seemed not to discriminate between the ‘I’ and ‘Me’ dimensions. Because electrode implantation sites are chosen based solely on clinical criteria, electrode coverage of the DN and rAI was not optimal, and only one recording site with positive results could be obtained in each of the three regions explored. Last, all recording sites tested were away from the epileptogenic regions, and did not include epileptic spikes, but more subtle signs of epileptic activity might have gone unnoticed. Despite these pitfalls, there is an overall good agreement between the MEG and iEEG data, as discussed further below, which suggests that MEG localization was rather accurate, and that epileptic patients performed the task in a similar manner as healthy participants.

Another caveat when working on HERs is that cardiac activity can generate two types of artefacts. The cardiac artefact corresponds to the contamination of neural data by the electrical signal of the heart. We analysed time windows that are devoid of this artefact [28] for both MEG and iEEG data, and further corrected MEG data using independent component analysis. The cardiac artefact appeared well suppressed from iEEG data once bipolar derivations are computed (see shaded areas in figures 3*a*, 4*a* and 5*g*). iEEG data are also susceptible to the pulse-related artefact [36] that appears as a slow frequency sinewave or sawtooth pattern. Given the transient nature of the effects reported here in iEEG data, as well as the good agreement between MEG and iEEG latencies and effect durations, it seems unlikely that the pulse-related artefact contributed to the iEEG results.

We also compared the electrophysiological results obtained with iEEG and MEG with MRI results from the literature. MEG source localization is performed on the grey matter ribbon, as can be seen in figure 5*e* and is expressed as a surface. MRI parcellations and functional regions involved in autonomous regulations are expressed in volumes. The conversion between volumes and surfaces might have generated some spatial noise.

## 6. Heartbeat evoked responses in the default network encode self-relatedness

As in healthy participants, iEEG recordings in epileptic patients show that HERs in the two midline nodes of the DN encoded self-relatedness. Intracranial data in single patients thus confirm the group-level source localization of MEG data in healthy participants [25]. Note that iEEG data confirm that neural responses to heartbeats in vPrC/vPCC are specific to the agentive ‘I’, but the high correlation between ‘I’ and ‘Me’ ratings of the patient implanted in vmPFC does not allow us to tease apart the two dimensions of the self in vmPFC. iEEG data also confirm the temporal order of the effects, with the effect in vmPFC appearing before the effect in vPrc/vPCC. iEEG data further extend the link between HERs and self-relatedness ratings down to the level of single trials, with significant correlations between trial-by-trial HER amplitude and self-relatedness of thought.

The detailed anatomical analysis of both iEEG and MEG source-localized results indicates that in vmPFC, the most active regions are areas 14 m and 32 [33], in the ventral part of the anterior cingulate cortex. iEEG recording sites located more laterally or more ventrally did not show any significant effect. Areas 14 m and 32 also contribute to sympathetic regulation [34]. In the posteromedial cortex, HERs varying with self-relatedness occurred in the vPrc and vPCC [32], that are not involved in autonomic regulation, as opposed to the area lying just anterior to them, that is associated with parasympathetic regulation. This result shows that regions that are not associated with autonomic regulation can nevertheless receive and differentially respond to cardiac information, depending on self-relatedness.

Our results thus confirm that the link between the self and DN [18] is expressed in neural responses to heartbeats, and directly support theories grounding the self in the monitoring of internal signals [1–3].

## 7. Heartbeat evoked responses in the right anterior insula contribute to encoding the ‘I’

Although a whole-brain analysis of MEG data revealed significant results only in the DN, a targeted ROI approach of the three sub-divisions of the insula revealed that neural responses to heartbeats in both the dorsal and ventral rAI vary according to the involvement of the ‘I’ in spontaneous thoughts, around 400 ms after the R-peak. Note that the effect was smaller in the rAI than in the DN, and appeared to stem from two distinct foci, which may explain why it was not detected in the whole-brain analysis despite a similar sensitivity of MEG to midline DN nodes and insula [37]. Intracranial recordings targeted the most anterior focus of the rAI in two patients. The effect could be detected in one patient out of two only.

These differential responses to heartbeats occurred during a resting state, without any explicit interoceptive task, because participants are not asked to orient attention to their heartbeats. We thus do not know whether these self-related neural responses to heartbeats in the rAI are linked to the modulation of neural responses to heartbeats in explicit interoceptive tasks. The location of the posterior focus in the rAI where we find differential HERs (figure 5*e*) is compatible with the meta-analysis of interoceptive tasks reported in this issue [38]. Still, it should be noted that explicit interoceptive tasks are likely to tap more onto the ‘Me’ dimension of the self (thinking about oneself) than about the agentive ‘I’ dimension of the self that we find to be encoded by HERs in the rAI both in MEG and iEEG data. Because rAI is involved in many cognitive studies [10,11] and is not specific to the self, as shown in the meta-analysis presented in figure 2, understanding the contribution of rAI to the self will certainly require further investigations [39].

## 8. Interplay between vmPFC, rAI, vPrc/vPCC and autonomic control regions

It has sometimes been proposed that the anterior insula is the cortical interoceptive hub, distributing interoceptive information to other cortical areas [3,40]. Our results rather speak in favour of multiple ascending pathways, as described in Critchley [41], and show a stronger effect in the DN than in the rAI. The earliest effects are observed around 400 ms after R peak, in overlapping time windows, i.e. almost simultaneously in the vmPFC and rAI, where the ‘Me’ and the ‘I’ self-dimensions are, respectively, encoded. This is in line with known direct projections from subcortical visceral relays to both insula and ventral cingulate regions [42]. The effect in the vPrc/vPCC corresponds to the same self-dimension as in the rAI, but appears later, around 580 ms after R peak, and is more robust. Because vPrc is connected to rAI [32], the weak rAI effect might fuel the more robust vPrc differential response. Alternatively, the vPrc/vPCC effect might be mediated through vmPFC, because the two structures are strongly functionally coupled [32]. In this case, it remains to be explained how the same cardiac inputs can give rise to the encoding of two different dimensions of the self in vPrc/vPCC and vmPFC.

In addition, our results suggest that self-related HERs correspond to a neural monitoring of cardiac information that does not directly translate into cardiac regulation. Indeed, there was no cardiac rhythm changes associated with the effects reported here. In addition, HER locations, that reflect the central monitoring of ascending cardiac information, do not map perfectly on regions involved in autonomic control, that reflect descending regulatory influences. In particular in the posterior medial cortex, the cortical territory involved in high-frequency cardiac regulation is distinct from the two adjacent regions, the ventral precuneus and vPCC, that show self-related HERs. It might be that the cortical cardiac monitoring function, initially devoted to autonomic regulation, has further evolved into a partially distinct process related to selfhood.

## 9. Conclusion

We here show that the amplitude of neural responses to heartbeats covaries with the self in both the DN and the rAI, although effects are weaker in the rAI. This implies that the literature on the self and DN should consider neural responses to heartbeats, and that conversely the literature relating interoception and the self in the rAI should consider the DN: both structures are related to the self through the same underlying mechanism.

## Supplementary Material

Supplementary Material, Figures and Tables

## Data accessibility

The anonymized MEG and iEEG data presented in the article can be obtained upon request to the first and last authors.
