# Supplementary material for: Is the cardiac monitoring function related to the self in both the default network and right anterior insula?
Source: Philos Trans R Soc Lond B Biol Sci. 2016 Nov 19;371(1708):20160004. doi: 10.1098/rstb.2016.0004 (PMC5062094; doi:10.1098/rstb.2016.0004)
Supplement: Supplementary Material, Figures and Tables [file rstb20160004supp1.docx]

**Supplementary material**

**Control on the cardiac field artefact on MEG data**

It is known that MEG and EEG signals are contaminated by the cardiac field artefact [1]. We therefore tested whether the ECG signal differed between “high” and “low” trials on the “I” scale, to check that the effects observed on MEG data were not reflecting a difference in the electrical activity of the heart picked up by the MEG sensors. The ECG signal did not differ (paired *t*-test, “high” vs. “low”, mean ECG amplitude averaged between 387 and 428 ms after the R-peak at each horizontal derivation, all |t(15)| < 0.81, all *p* > 0.43; at each vertical derivation, all |t(15)| < 0.95, all *p* > 0.36).

This test could not be performed in patients. Although R-peaks were clearly visible in the ECG, the overall level of noise was quite high. However, the cardiac field artefact should be strongly attenuated by the computation of bipolar derivation. Besides, the cardiac-field artefact is not present in the time-window analyzed (300-600 ms post R-peak).

**Supplementary Figures**

**
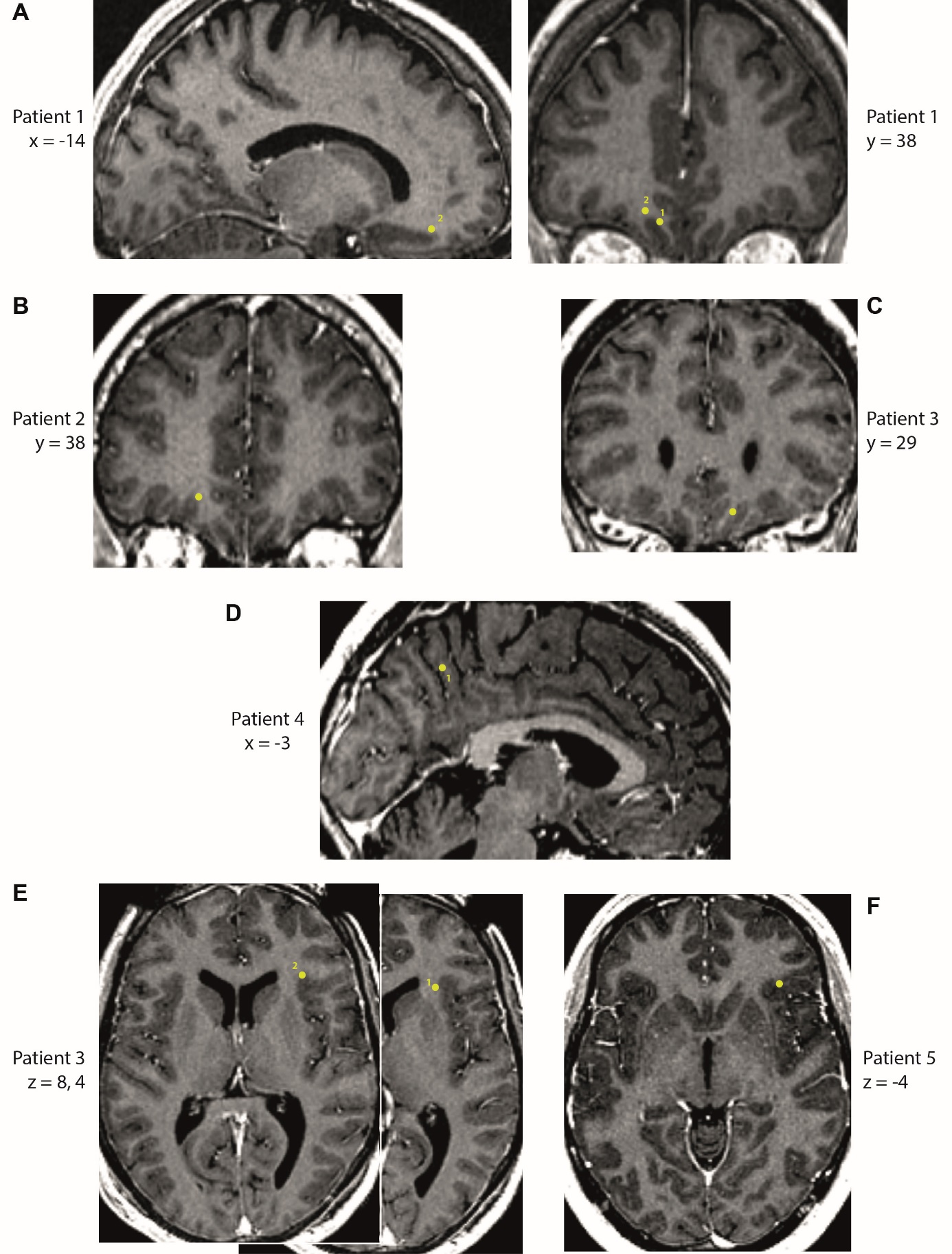
**

**Supplementary Figure 1: Individual MRIs of the patients, normalized to MNI space**

A, Sagittal (left, x=-14) and coronal (right, y=38) views of the MRI of patient 1, showing the recording sites of the electrode shaft located in the vmPFC region.

B, C, Coronal views (y=38, y=29) of the MRIs of patients 2 and 3, respectively, showing the two recording sites in the vmPFC.

D, Sagittal view (x=-3) of the MRI of patient 4, showing the most medial recording site analyzed (recording site 1).

E, Axial views (top: z=8, bottom: z=4) of the MRI of patient 3, showing the two recording sites located in the insular cortex

F, Axial view (z=-4) of the MRI of patient 5, showing the two recording sites in the insular cortex.


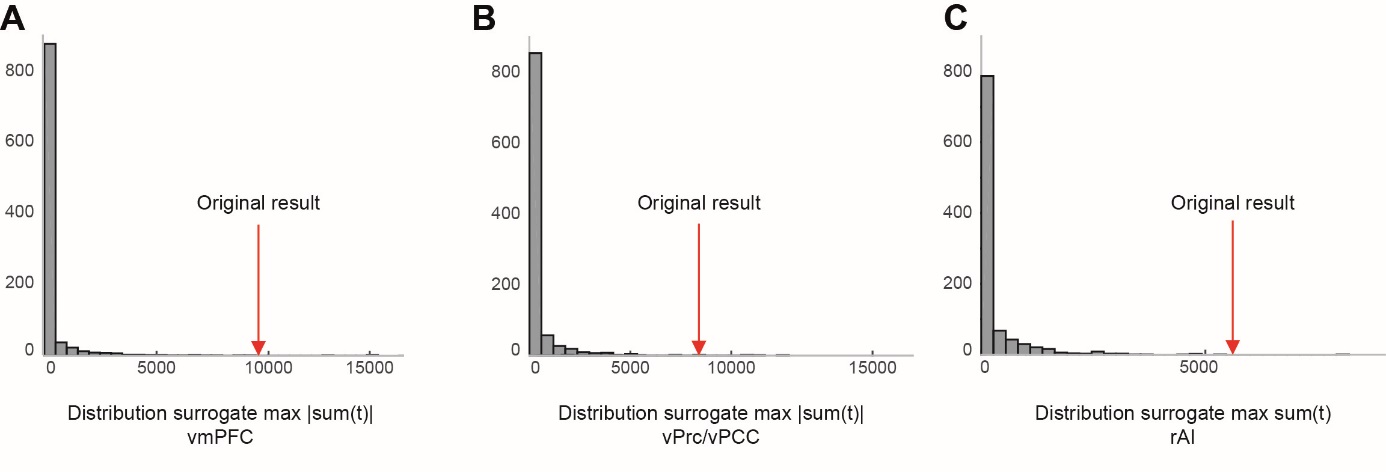


**Supplementary Figure 2: Test on 1000 surrogate heartbeats**

Histograms of the distribution of the maximal cluster *t* statistic obtained for the 1000 permutations of surrogate heartbeats, for iEEG data obtained in recording site 2 of patient 1 (vmPFC, A), recording site 1 of patient 4 (vPrc/vPCC, B) and recording site 2 of patient 5 (rAI, C). The original cluster *t* statistics (red arrows) lie in the tail of the distributions, indicating that the reported effects are truly locked to heartbeats.

**Supplementary tables**

**Supplementary table 1:** Information about intracranially recorded patients.

| **Patient** | **1** | **2** | **3** | **4** | **5** |
| --- | --- | --- | --- | --- | --- |
| **Age** | 39 | 23 | 25 | 30 | 21 |
| **Gender** | F | M | F | M | F |
| **Educational level / IQ** | Bachelor degree  IQ = 106 | 2 years of higher education  IQ = 91 | Master degree | 2 years of higher education | Bachelor degree |
| **Epilepsy duration (years)** | 16 | 20 | 12 | 12 | 13 |
| **Epilepsy focus** | Left inferior temporal gyrus | Left middle/inferior temporal pole | Right middle temporal gyrus / right hippocampus | Left supplementary motor area | Right anterior/mid cingulate area |
| **Recording system** | Neuralynx | Micromed | Neuralynx | Micromed | Neuralynx |
| **Number of clean trials / Total number of trials** | 27 / 27 | 33 / 36 | 26 / 27 | 45 / 45 | 24 / 27 |
| **Scales analyzed** | “Me”  Mean: 115±11  Median: 133 | “Me”  Mean: 101±6  Median: 102 | vmPFC: “Me”  Mean: 83±12  Median: 72  Insula: “I”  Mean: 93±15  Median: 66 | “I”  Mean: 96±12  Median: 100 | “I”  Mean: 109±18  Median: 121 |
| **Pearson correlation coefficient r, between “I” and “Me” ratings** | 0.91 | 0.70 | 0.84 | 0.13 | 0.70 |
| **Interbeat interval (ms)** | 881 | 771 | 978 | 1017 | 681 |

**Supplementary table 2**: anatomical description of the regions present in the reverse inference meta-analysis on the term “self”, based on Automated Anatomical Labeling (AAL) atlas [2]. Only areas with more than 3% of their volume involved are listed.

| AAL region | % activation | mm^3^ | peak z | z/mm^3^ | MNI | | |
| --- | --- | --- | --- | --- | --- | --- | --- |
|  |  |  |  |  | X | Y | Z |
| Left medial orbitofrontal gyrus | 28.40 | 204 | 7.70 | 4.86 | -8 | 46 | -10 |
| Left posterior cingulate gyrus | 24.80 | 115 | 6.17 | 4.60 | -4 | -52 | 32 |
| Left superior frontal gyrus, medial | 22.30 | 666 | 8.73 | 5.06 | -4 | 54 | 4 |
| Left anterior cingulate and paracingulate gyri | 19.50 | 273 | 7.28 | 4.79 | -10 | 50 | 2 |
| Right posterior cingulate gyrus | 19.10 | 64 | 6.18 | 4.64 | 2 | -52 | 30 |
| Right medial orbitofrontal gyrus | 15.40 | 132 | 5.31 | 4.34 | 6 | 48 | -8 |
| Right angular gyrus | 10.00 | 176 | 6.81 | 4.63 | 50 | -58 | 28 |
| Left precuneus | 9.58 | 338 | 7.22 | 4.58 | -2 | -58 | 18 |
| Left angular gyrus | 8.95 | 105 | 5.97 | 4.21 | -48 | -66 | 32 |
| Right superior frontal gyrus, medial | 8.06 | 172 | 7.46 | 4.58 | 2 | 54 | 22 |
| Right gyrus rectus | 6.98 | 52 | 5.19 | 4.25 | 4 | 44 | -16 |
| Left superior frontal gyrus, dorsolateral | 6.53 | 235 | 6.56 | 4.42 | -8 | 70 | 16 |
| Left temporal pole, middle temporal gyrus | 6.09 | 46 | 5.87 | 4.51 | -52 | 14 | -34 |
| Right precuneus | 3.52 | 115 | 5.39 | 4.30 | 4 | -52 | 24 |

**Supplementary table 3**: anatomical description of the regions present in the forward inference meta-analysis on the term “self”, based on Automated Anatomical Labeling (AAL) atlas [2]. Only areas with more than 3% of their volume involved are listed.

| AAL region | % activation | mm^3^ | peak z | z/mm^3^ | MNI | | |
| --- | --- | --- | --- | --- | --- | --- | --- |
|  |  |  |  |  | X | Y | Z |
| Left amygdala | 88.60 | 195 | 16.10 | 8.13 | -22 | -4 | -16 |
| Right amygdala | 63.70 | 158 | 15.10 | 7.85 | 22 | -2 | -16 |
| Left lenticular nucleus, pallidum | 59.70 | 175 | 11.20 | 5.37 | -14 | 8 | -4 |
| Left anterior cingulate and paracingulate gyri | 59.10 | 827 | 12.30 | 5.39 | -6 | 46 | -4 |
| Left angular gyrus | 55.80 | 655 | 14.20 | 5.63 | -48 | -66 | 32 |
| Left insula | 53.40 | 992 | 23.80 | 7.73 | -34 | 20 | 0 |
| Right lenticular nucleus, pallidum | 51.40 | 144 | 9.34 | 5.23 | 14 | 6 | 0 |
| Left posterior cingulate gyrus | 51.40 | 238 | 13.40 | 7.05 | 0 | -54 | 28 |
| Left medial orbitofrontal gyrus | 51.30 | 369 | 13.20 | 6.63 | -4 | 46 | -8 |
| Right inferior parietal, excluding  supramarginal and angular gyri | 49.10 | 661 | 10.70 | 4.80 | 50 | -38 | 48 |
| Right inferior frontal gyrus, opercular part | 44.10 | 617 | 11.80 | 5.31 | 50 | 10 | 28 |
| Left superior frontal gyrus, medial | 44.10 | 1318 | 15.90 | 5.98 | 2 | 24 | 44 |
| Right insula | 43.20 | 765 | 19.70 | 7.71 | 34 | 22 | -4 |
| Left inferior frontal gyrus, opercular part | 41.30 | 429 | 12.60 | 5.20 | -44 | 6 | 28 |
| Left thalamus | 40.90 | 450 | 11.00 | 5.29 | -10 | -16 | 8 |
| Left inferior parietal, excluding  supramarginal and angular gyri | 40.60 | 993 | 8.52 | 4.50 | -34 | -56 | 44 |
| Right caudate nucleus | 38.90 | 387 | 10.40 | 5.41 | 10 | 10 | 0 |
| Left inferior frontal gyrus, triangular part | 38.30 | 969 | 13.70 | 4.65 | -38 | 22 | -2 |
| Left supplementary motor area | 37.50 | 805 | 17.80 | 7.33 | -2 | 14 | 50 |
| Right anterior cingulate and paracingulate gyri | 36.70 | 482 | 9.34 | 4.67 | 4 | 32 | 28 |
| Left hippocampus | 36.60 | 341 | 13.20 | 5.56 | -20 | -10 | -16 |
| Right medial orbitofrontal gyrus | 34.90 | 299 | 9.89 | 5.05 | 2 | 48 | -8 |
| Left lenticular nucleus, putamen | 34.70 | 350 | 11.50 | 4.88 | -14 | 10 | -4 |
| Right angular gyrus | 34.20 | 600 | 11.00 | 4.73 | 50 | -58 | 26 |
| Left inferior frontal gyrus, orbital part | 33.10 | 560 | 13.20 | 5.62 | -34 | 22 | -12 |
| Right posterior cingulate gyrus | 32.20 | 108 | 14.00 | 5.85 | 2 | -50 | 28 |
| Left precentral gyrus | 31.80 | 1121 | 12.60 | 4.99 | -46 | 6 | 32 |
| Right inferior frontal gyrus, triangular part | 30.30 | 651 | 10.70 | 4.55 | 50 | 22 | 4 |
| Right hippocampus | 27.70 | 262 | 11.20 | 5.35 | 22 | -2 | -20 |
| Right thalamus | 27.20 | 287 | 7.44 | 4.37 | 10 | -18 | 4 |
| Right lenticular nucleus, putamen | 27.10 | 288 | 9.34 | 4.39 | 30 | 18 | 2 |
| Right median cingulate and paracingulate gyri | 25.20 | 556 | 13.70 | 5.59 | 4 | 18 | 44 |
| Left median cingulate and paracingulate gyri | 23.20 | 450 | 11.80 | 5.20 | -2 | 14 | 42 |
| Left supramarginal gyrus | 23.20 | 291 | 6.35 | 3.95 | -54 | -26 | 20 |
| Right superior frontal gyrus, medial | 23.10 | 493 | 15.10 | 5.47 | 4 | 24 | 44 |
| Left precuneus | 22.30 | 787 | 13.40 | 5.96 | -2 | -56 | 20 |
| Left caudate nucleus | 21.40 | 206 | 9.89 | 4.62 | -10 | 10 | -2 |
| Right supplementary motor area | 21.30 | 506 | 13.20 | 5.97 | 6 | 22 | 46 |
| Right supramarginal gyrus | 19.30 | 380 | 8.80 | 4.07 | 48 | -38 | 44 |
| Left middle temporal gyrus | 19.10 | 943 | 9.34 | 4.46 | -52 | -58 | 20 |
| Left rolandic operculum | 17.80 | 176 | 8.52 | 4.21 | -48 | 6 | 4 |
| Right inferior frontal gyrus, orbital part | 16.80 | 286 | 15.90 | 5.96 | 36 | 22 | -8 |
| Left superior parietal gyrus | 14.80 | 305 | 6.89 | 3.98 | -30 | -60 | 44 |
| Left inferior occipital gyrus | 14.00 | 132 | 6.35 | 3.92 | -42 | -74 | -4 |
| Right superior parietal gyrus | 13.50 | 301 | 7.71 | 4.34 | 26 | -62 | 56 |
| Right parahippocampal gyrus | 13.40 | 152 | 8.52 | 4.12 | 20 | -2 | -20 |
| Left parahippocampal gyrus | 13.00 | 127 | 7.44 | 3.83 | -14 | -4 | -18 |
| Right precuneus | 12.20 | 398 | 11.80 | 4.82 | 2 | -54 | 28 |
| Right precentral gyrus | 11.90 | 402 | 14.00 | 4.93 | 50 | 8 | 32 |
| Right olfactory cortex | 11.80 | 34 | 6.07 | 3.93 | 4 | 12 | -4 |
| Right middle temporal gyrus | 11.60 | 510 | 7.71 | 4.02 | 58 | -8 | -18 |
| Left postcentral gyrus | 11.30 | 440 | 8.52 | 4.28 | -38 | -22 | 54 |
| Left middle occipital gyrus | 10.70 | 350 | 11.00 | 4.30 | -46 | -70 | 4 |
| Left superior frontal gyrus, dorsolateral | 8.28 | 298 | 7.71 | 4.27 | -24 | -4 | 54 |
| Right gyrus rectus | 8.19 | 61 | 8.52 | 4.84 | 2 | 50 | -16 |
| Right inferior occipital gyrus | 8.09 | 80 | 5.53 | 3.86 | 38 | -86 | -4 |
| Left gyrus rectus | 7.98 | 68 | 8.25 | 4.82 | -2 | 46 | -16 |
| Left middle frontal gyrus | 7.77 | 378 | 8.52 | 4.04 | -26 | -2 | 56 |
| Right middle frontal gyrus | 7.46 | 381 | 6.62 | 3.74 | 28 | -2 | 52 |
| Left calcarine fissure and surrounding cortex | 7.44 | 168 | 6.89 | 4.16 | -14 | -50 | 4 |
| Right superior temporal gyrus | 7.29 | 229 | 7.71 | 3.86 | 54 | -58 | 22 |
| Right middle occipital gyrus | 6.34 | 133 | 6.89 | 3.63 | 46 | -74 | 4 |
| Right rolandic operculum | 5.56 | 74 | 7.16 | 4.03 | 52 | 10 | 0 |
| Right superior occipital gyrus | 4.39 | 62 | 5.53 | 3.87 | 32 | -64 | 40 |
| Left superior temporal gyrus | 4.36 | 100 | 5.53 | 3.63 | -50 | -20 | 12 |
| Left fusiform gyrus | 4.20 | 97 | 6.35 | 3.75 | -28 | -40 | -16 |
| Left temporal pole, superior temporal gyrus | 3.66 | 47 | 7.16 | 4.31 | -50 | 6 | 0 |
| Right hemispheric lobule VI (cerebelum) | 3.57 | 64 | 4.44 | 3.48 | 28 | -58 | -24 |
| Right fusiform gyrus | 3.38 | 85 | 4.71 | 3.49 | 28 | -30 | -18 |
| Left olfactory cortex | 3.21 | 9 | 4.17 | 3.59 | -2 | 22 | -6 |

**Supplementary table 4**: iEEG recording sites of interest in the vmPFC region. The recording site showing a significant effect is indicated by a star. The distance between a recording site and a region of interest corresponds to the minimal distance between the recording site and all the voxels belonging to the region.

| **Patient** | **1** | | **2** | **3** |
| --- | --- | --- | --- | --- |
| **Recording site number** | 1 | 2* | 1 | 1 |
| **MNI coordinates** | -9 38 -20 | -14 38 -16 | -18 38 -12 | 10 29 -19 |
| **Distance to MEG cluster (mm)** | 9 | 9 | 10 | 11 |
| **Distance to region 14m (mm)** | 4 | 5 | 7 | Inside right 14m |
| **Distance to region 32 (mm)** | 13 | 8 | 6 | / |

**Supplementary table 5**: iEEG recording sites of interest in the vPrc region. The recording site showing a significant effect is indicated by a star. The distance between a recording site and a region of interest corresponds to the minimal distance between the recording site and all the voxels belonging to the region.

| **Patient** | **4** | |
| --- | --- | --- |
| **Recording site number** | 1* | 2 |
| **MNI coordinates** | -3 -53 49 | -7 -55 52 |
| **Distance to MEG cluster (mm)** | 15 | 17 |
| **Distance to vPrc (mm)** | Inside | 2 |

**Supplementary table 6**: iEEG recording sites of interest in the anterior insula region. The recording site showing a significant effect is indicated by a star. The distance between a recording site and a region of interest corresponds to the minimal distance between the recording site and all the voxels belonging to the region.

| **Patient** | **3** | | **5** |
| --- | --- | --- | --- |
| **Recording site number** | 1 | 2* | 1 |
| **MNI coordinates** | 28 25 4 | 32 27 8 | 36 28 -4 |
| **Distance to dAI (mm)** | 4 | 4 | 5 |
| **Distance to vAI (mm)** | 10 | 14 | 7 |

**Supplementary Table 7**: additional information about MEG recordings, from 16 healthy participants.

|  | **“I” scale** | **“Me” scale** |
| --- | --- | --- |
| **Median ± SEM** | 135.4±12.4 | 84.7±11.5 |
| **Average number of clean trials ± SEM**  **(total number of trials: 80)** | High:40.4±0.2  Low:38.9±0.3 | High:40.1±0.2  Low:39.1±0.3 |
| **Mean Pearson correlation coefficient**  **between “I” and “Me” ratings (±s.e.m.)** | r=0.67±0.04 | |

**Supplementary References**

1. Kern, M., Aertsen, A., Schulze-Bonhage, A. & Ball, T. 2013 Heart cycle-related effects on event-related potentials, spectral power changes, and connectivity patterns in the human ECoG. *Neuroimage* **81**, 178–90. (doi:10.1016/j.neuroimage.2013.05.042)

2. Tzourio-Mazoyer, N., Landeau, B., Papathanassiou, D., Crivello, F., Etard, O., Delcroix, N., Mazoyer, B. & Joliot, M. 2002 Automated anatomical labeling of activations in SPM using a macroscopic anatomical parcellation of the MNI MRI single-subject brain. *Neuroimage* **15**, 273–289. (doi:10.1006/nimg.2001.0978)
